# Tumors of the Lacrimal Drainage System: Diagnosis and Management

**DOI:** 10.3390/cancers18121894

**Published:** 2026-06-10

**Authors:** Ernest Iakovlev, Kaveh Vahdani, David H. Verity, Simon Gane, Geoffrey E. Rose

**Affiliations:** 1 Orbital Service, Moorfields Eye Hospital, London EC1V 2PD, UK; 2 Royal National ENT and Eastman Dental Hospitals, London WC1E 6DG, UK

**Keywords:** lacrimal drainage system, lacrimal sac, nasolacrimal duct, lacrimal sac tumors, orbit, tumor, epiphora, dacryocystitis

## Abstract

Tumors of the lacrimal drainage system are rare and include benign and malignant variants. Their presentation frequently mimics inflammatory or obstructive lacrimal disease, which often results in delayed diagnosis and diagnostic uncertainty. A high index of suspicion, appropriate imaging, and timely biopsy are required to verify the histology and guide management. Surgical excision remains the cornerstone of treatment for most lesions, with the aim of achieving histologically clear margins. Advances in endoscopic and combined surgical approaches have enabled radical tumor resection whilst reducing morbidity and improving globe preservation. Adjuvant radiotherapy is commonly employed in malignant disease, while systemic chemotherapy, immunotherapy, and targeted therapies may be considered in selected advanced or recurrent cases. Emerging molecular insights and evolving surgical techniques may further refine future treatment strategies. This review summarizes current evidence and clinical principles to provide a practical framework for the diagnosis and management of lacrimal drainage system tumors.

## Simple Summary

Lacrimal drainage system tumors are rare, can be benign or malignant, and often cause symptoms resembling common conditions—such as blocked tear ducts or infections—which can make early recognition difficult. Complete surgical excision is currently the treatment of choice, but newer surgical techniques, including minimally invasive and endoscopic approaches, might reduce complications and help preserve ocular function. Radiotherapy, chemotherapy, and/or targeted therapies may be used as adjuvant or neo-adjuvant therapy for malignant tumors. This review summarizes current evidence and clinical principles, as well as new research, to provide a practical framework for the diagnosis and management of lacrimal drainage system tumors.

## 1. Introduction

Tumors of the lacrimal drainage system (LDS) are rare, histologically diverse (with several benign and malignant variants), and mainly originate in the lacrimal sac or nasolacrimal duct [1,2]. Symptoms often mimic benign lacrimal disease, such as chronic dacryocystitis or nasolacrimal duct obstruction [3] and can result in delayed investigation and detection of malignancy [1,2,3].

Management of LDS tumors is complex and requires multidisciplinary care tailored to histology. Complete excision with clear margins remains the cornerstone of treatment, and local surgical approaches can permit globe-sparing approaches [1,2,3,4,5,6,7]. Owing to their rarity, management largely relies on retrospective series and extrapolation from sinonasal oncology. This narrative review summarizes the current evidence and institutional experience of LDS tumors, including histopathology, presentation, diagnosis, and management.

## 2. Histopathological Classification

Primary LDS tumors are classified by cell of origin—epithelial, mesenchymal, lymphoid, melanocytic, or neural (Table 1)—and arise predominantly in the lacrimal sac [1,2,3]. Primary epithelial tumors are the most common LDS neoplasms [1,2], and benign papillomas, although generally not reported, are the most common lesions of the lacrimal sac [1,3]. LDS may also be affected by tumors arising in adjacent structures—such as the orbit, paranasal sinuses, or skin—or, rarely, by metastases. Non-neoplastic processes (Table 2), such as chronic inflammation or granulomatous disease, can also present with a lacrimal sac mass [1].

Epithelial tumors arise from the pseudostratified ciliated columnar epithelium that lines LDS. Papillomas are the most common benign epithelial tumors and may be exophytic (expanding into the sac lumen) or endophytic (growing deeply, expanding the epithelial basement membrane) [1,3]. Endophytic (inverted) papillomas are associated with higher recurrence rates and an estimated 10–15% risk of malignant transformation, which can be either synchronous (found at the time of primary surgery) or metachronous (occurring many years later). The timeline for malignant transformation is variable, and long-term surveillance is therefore recommended [8,9].

Malignant epithelial tumors account for most primary LDS malignancies [10], the most common being squamous cell carcinoma (SCC)—which may arise de novo or from benign papillomas—but other variants include transitional-cell carcinoma or adenoid cystic carcinoma [1,2,3,10]; adenoid cystic carcinoma being characterized by aggressive behavior and a propensity for perineural invasion. Rare epithelial subtypes, such as mucoepidermoid carcinoma, adenocarcinoma, and oncocytic carcinoma, have also been described [1,2,3,10].

Non-epithelial tumors may arise from connective tissue, lymphoid elements, or melanocytes. Lymphomas, typically extranodal marginal zone lymphomas, are the most common non-epithelial LDS malignancy, but other more aggressive variants—such as diffuse large B-cell lymphoma—can affect LDS [2,3].

Primary malignant melanoma of LDS is extremely rare and often amelanotic [11]. Mesenchymal tumors are uncommon and include lesions such as solitary fibrous tumor, fibrous histiocytoma, lipoma, and cavernous venous malformation [1].

Primary punctal and canalicular neoplasms are rare. Benign lesions include melanocytic naevi, papillomas, hidrocystoma, and epidermoid cysts, whereas malignant tumors include squamous cell carcinoma, sebaceous carcinoma, melanoma, and lymphoma. Secondary involvement may occur through extension of adjacent eyelid, conjunctival, or lacrimal sac malignancies [12,13].

## 3. Etiology and Risk Factors

LDS tumors are largely idiopathic, although several risk factors have been proposed, including chronic inflammation that might promote epithelial metaplasia and subsequent neoplasia [3]. Human papillomavirus (HPV) infection has also been implicated in epithelial tumors, with low-risk subtypes (HPV-6, HPV-11) associated with papillomas and high-risk subtypes (HPV-16, HPV-18) linked to carcinomas [14]. Malignant transformation may occur within pre-existing papillomas, particularly those of the endophytic subtype, and has also been reported following irradiation of benign papillomas [2].

## 4. Epidemiology and Demographics

The various LDS tumors have different typical ages at presentation: benign epithelial and mesenchymal tumors usually present earlier, whereas malignant tumors occur more often in middle-aged and older adults [1,2,3,8,10]. Likewise, whilst most papers report no gender predilection for epithelial tumors, some case series found carcinomas (particularly squamous) to have a slight male predominance (55–57%), whereas adenoid cystic carcinoma has a female predominance (76–80%) [1,2,3,6,10].

Lymphomas are the most common in women (~60%) and usually present in the sixth decade [1,3,15]. Primary mucosal melanomas of LDS show no gender predilection and typically present in the sixth decade [11].

## 5. Clinical Presentation

**Figure 1:**
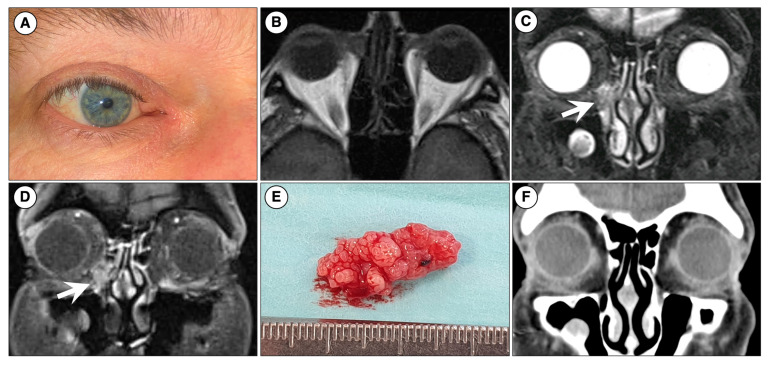
A 52-year-old man presenting with a year of right-sided epiphora, a single episode of dacryocystitis, and intermittent hemolacrima. (**A**) Examination showed upper and lower canaliculitis with granulomatous tissue within the upper canaliculus. MRI demonstrated an enlarged right lacrimal sac, hypointense on T1 (**B**), heterogeneous on T2 fat-suppressed sequences (**C**), and with moderate post-contrast enhancement (**D**). (**E**) Surgical exploration revealed a papillomatous lesion involving the lacrimal sac and canalicular system. Dacryocystectomy with upper and lower canaliculotomy was performed, together with fulguration of the nasolacrimal duct. Histology confirmed a benign exophytic squamous papilloma. (**F**) CT at 6 years showed no recurrence.

**Figure 2:**
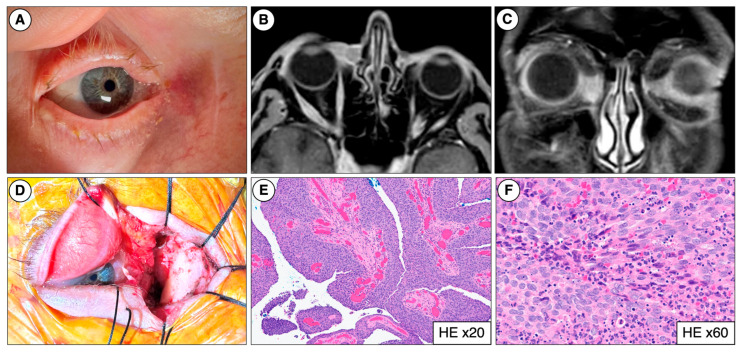
Four years after right dacryocystectomy for an inflamed, inverted transitional-cell papilloma, a 78-year-old woman presented with an upper canalicular mass, medial canthal pain, ocular discharge, and erythema (**A**). MRI showed a heterogeneously enhancing lesion, centered on the upper canaliculus, with extension into the lacrimal sac fossa (**B**,**C**). En bloc resection of the mass, the canaliculi, and the adjacent bulbar conjunctiva achieved macroscopic clearance (**D**). Histopathology showed a transitional-cell lesion with papillary fronds and focal stromal invasion (**E**), with frequent mitotic figures and areas of necrosis (**F**), consistent with non-keratinizing squamous cell carcinoma arising in residual transitional-cell papilloma (carcinoma ex-papilloma). Tumor cells were negative for p16 and HPV-RNA in situ hybridization; RNA-based next-generation sequencing identified a DEK::AFF2 fusion. Subsequent endoscopic resection of the nasolacrimal duct showed no tumor involvement.

**Figure 3:**
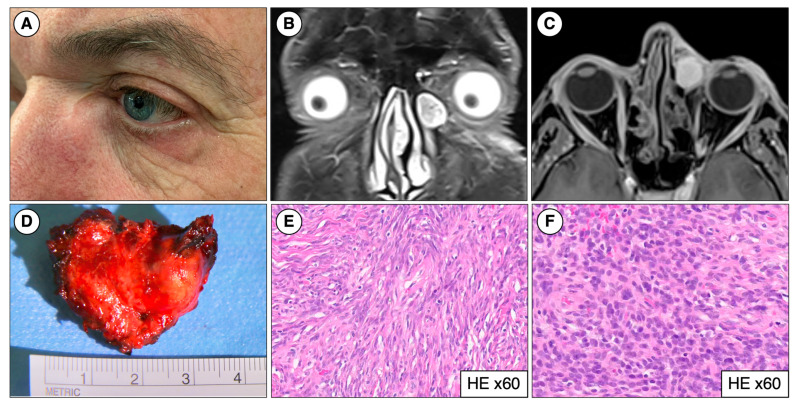
A 52-year-old man with 5 years of left-sided epiphora and a painless, non-compressible medial canthal mass for a year, this mass extending above the medial canthal tendon (**A**). (**B**) MRI demonstrated a well-circumscribed lesion with high T2 signal (**B**) and avid post-contrast enhancement (**C**). The lesion was excised intact (**D**), and histopathology showed a completely excised low-grade solitary fibrous tumor composed of spindle-shaped and ovoid cells (**E**,**F**) with characteristic branching (“staghorn”) vasculature and focal hyalinized collagenous stroma. Tumor cells were CD34- and STAT6-positive, and the nasolacrimal duct was free of tumor.

**Figure 4:**
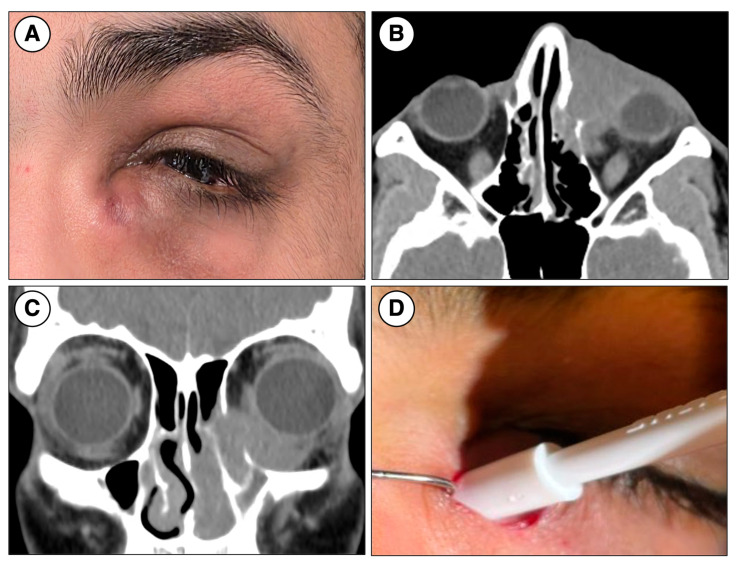
(**A**) A firm and painless left medial canthal mass, extending into the lower eyelid and above the canthal tendon, was evident in a 32-year-old patient with 4 months of hemolacrima. (**B**,**C**) A relatively homogeneous hypodense lesion, centered on the lacrimal drainage system, was demonstrated on CT, together with bone erosion and extension into the medial orbit and sinonasal cavity. (**D**) Transcutaneous punch biopsy confirmed diffuse large B-cell lymphoma.

LDS tumors often present with epiphora and a medial canthal mass, relatively common symptoms that initially suggest dacryocystitis (Figure 1, Figure 2, Figure 3 and Figure 4). Lacrimal syringing may be normal [1,2,3,15].

Malignant lesions are often fixed, fairly firm, and non-reducible, and are usually painless unless complicated by infection or perineural invasion [2,16]; adenoid cystic carcinoma may present with pain or paresthesia. Whilst non-neoplastic conditions are still the most common cause of an LDS mass—whether above or below the medial canthal tendon—extension above the tendon strongly favors malignancy (Figure 3A and Figure 4A) [17]. Overlying skin changes—including ulceration, induration, discoloration, or fistula formation—suggest aggressive disease. Whilst commonly amelanotic, LDS melanomas may present as a light-purple lesion visible through conjunctiva or skin [11]. Hemolacrima may suggest malignancy, especially if occurring with epistaxis, suggesting sinonasal extension [3,11].

## 6. Diagnostic Evaluation

Unrecognized malignancy at lacrimal bypass surgery risks tumor seeding into the orbit and nasal cavity, thereby compromising tumor resection and prognosis. Atypical presentation of lacrimal drainage disease therefore warrants imaging and biopsy, and suspicious findings at dacryocystorhinostomy should prompt biopsy without osteotomy [1,3,17].

Computed X-ray tomography (CT), which delineates bony anatomy, is the first-line imaging modality. Benign lesions, such as large mucoceles, tend to cause bony expansion or remodeling of the lacrimal fossa, whereas malignant lesions often cause bone destruction (Figure 4B,C) [1,10]. Magnetic resonance imaging (MRI) provides soft-tissue detail, which can facilitate assessment of tumor extent, orbital invasion, perineural spread, and differentiation of tissue from retained secretions within LDS (Figure 1, Figure 2 and Figure 3) [1,3,10].

Dacryocystography (DCG) may demonstrate filling defects within the lacrimal drainage system and can help localize the level of obstruction. Modern hybrid techniques such as CT-DCG or MRI-DCG allow simultaneous evaluation of the intraluminal anatomy and surrounding soft tissues [18].

Tissue diagnosis is essential for suspected malignancy: palpable lesions should undergo transcutaneous incisional biopsy (Figure 4D), whilst incidental intraoperative masses warrant excisional biopsy without the creation of an osteotomy [1,17]. Should a tumor be confirmed, further investigations might include MRI of the head and neck, whole-body PET-CT (if malignancy), and cervical lymph node assessment to evaluate for regional or distant metastases [2].

Dacryoendoscopy can aid evaluation by enabling direct visualization of intraluminal lesions and facilitating targeted biopsy [19,20]. Emerging evidence suggests that circulating tumor DNA (ctDNA) may provide a non-invasive biomarker for detection and monitoring of HPV-associated lacrimal sac SCC, although further validation is required [21].

## 7. Management

**Figure 5:**
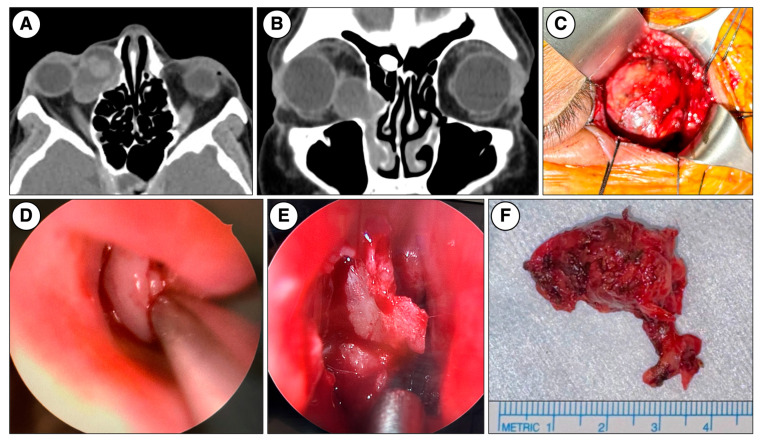
Combined external and endoscopic approach for en bloc resection of a non-keratinizing squamous cell carcinoma of the lacrimal drainage system in a 68-year-old woman with prior dacryocystorhinostomy and multiple resections for recurrent transitional-cell papillomas. (**A**,**B**) CT revealed a large, circumscribed lesion with heterogeneous contents, extending inferomedially into the right orbit and nasal cavity through the previous rhinostomy, along with enlargement of the right nasolacrimal duct. (**C**) External exposure of the tumor. (**D**) Endoscopic view of the inferior meatus showing tumor involvement. (**E**) A prelacrimal recess approach with removal of the medial bony wall of the nasolacrimal duct facilitated en bloc resection of the tumor (**F**).

Surgical excision with histologically clear margins remains the principal treatment modality for most LDS tumors. To minimize the risk of tumor seeding and recurrence, en bloc resection is generally recommended for malignant tumors and inverted papillomas (Figure 5) [1,22].

**Figure 6:**
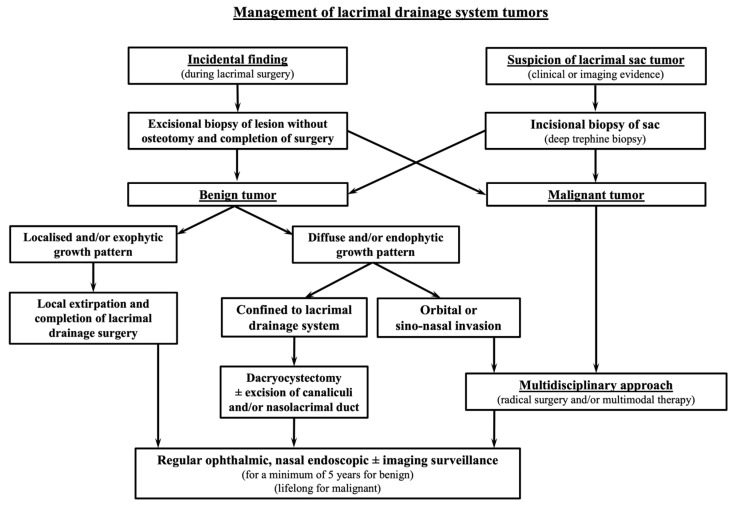
Proposed algorithm for management of lacrimal drainage system tumors, based on the current evidence and the authors’ experience. Suspected lacrimal sac tumors should undergo biopsy for histological confirmation. Management is determined by tumor type and extent. Localized benign lesions are treated with excision, while diffuse disease confined to the drainage system may require dacryocystectomy with canalicular and/or nasolacrimal duct excision. Tumors with orbital or sinonasal extension and all malignant lesions require multidisciplinary management, often combining surgery with radiotherapy and/or systemic therapy. Long-term clinical, endoscopic, and imaging surveillance is recommended.

The choice of surgery depends on tumor characteristics and extent, as well as patient factors. Localized benign tumors may be managed with complete excision and completion of lacrimal drainage surgery [1]. Dacryocystectomy—with resection of local periosteum and as much nasolacrimal duct as possible—is recommended for diffuse, invasive, or endophytic benign tumors; such resections are often accomplished through a tear trough, medially extended subciliary, or extended DCR incision—incorporating, if necessary, the canaliculi, involved skin, or any prior biopsy tract (Figure 6) [1]. These authors favor a lateral approach which involves a medial canthal incision to isolate the canaliculi, with a retrocaruncular conjunctival incision similar to that employed for a medial wall decompression. This approach allows complete exposure and isolation of the lesion, including those with a mass effect in the orbit. On closure, the resultant orbicularis flap tends to reduce the risk of postoperative fistula formation.

Dacryoendoscopy may facilitate minimally invasive management of selected intraluminal lesions, such as canalicular papilloma, and has been used to guide transcanalicular interferon-α2b therapy. However, its role in the treatment of LDS tumors remains limited and adjunctive [19,20].

Locally advanced malignancies involving the orbit or sinonasal structures may require extensive resection, including orbital walls and/or sinonasal structures. Globe-preservation is preferred where clear resection margins can be achieved but may be limited by the need for adjuvant therapy and treatment-related ocular morbidity. Orbital exenteration is reserved for those rare cases with extensive orbital involvement, extraocular muscle infiltration, scleral invasion, or a blind, painful eye [1,2,3,10]. Combined endonasal and external approaches, often incorporating limited external incisions, enable en bloc resection with reduced morbidity and are particularly suited to extensive disease or lesions with limited orbital extension [23,24]. Other approaches, including modified lateral rhinotomy, sublabial (midfacial degloving), and transnasal techniques, may be selected according to tumor extent, sinonasal involvement, and cosmetic considerations [5,25,26].

## 8. Adjuvant Therapies

Radiotherapy is commonly employed, particularly for epithelial carcinomas and melanoma, and is indicated for close or positive margins, perineural invasion, or poorly differentiated tumors; typical postoperative regimens deliver 50–60 Gy in 2 Gy fractions [11,22].

Neoadjuvant systemic chemoreductive therapy may be used to reduce the preoperative size of primary LDS carcinomas and facilitate eye-sparing tumor resections, and regimens include cisplatin or docetaxel combined with Programmed Cell Death Protein 1 (PD-1) or Epidermal Growth Factor Receptor (EGFR) inhibitors [27].

For primary LDS lymphomas, surgery is usually limited to diagnostic biopsy, with or without debulking. Definitive management is dependent on subtype: localized low-grade disease (for example, extranodal marginal zone lymphoma) responds well to external beam radiotherapy, whereas high-grade disease (for example, diffuse large B-cell lymphoma) is often disseminated and requires systemic immunochemotherapy (for example, “R-CHOP”—rituximab, cyclophosphamide, doxorubicin, vincristine, and prednisolone) [15].

Given the limited efficacy of conventional chemotherapy, management of melanomas increasingly incorporates targeted therapies, such as BRAF inhibitors, and/or immune checkpoint inhibitors (such as pembrolizumab) [11]. Emerging data support durable responses with combination immunotherapy, such as nivolumab with ipilimumab, in patients with advanced melanoma [28].

Intraoperative application of topical antimetabolites (0.02% mitomycin C or 1% 5-fluorouracil) for 3–5 min, or their postoperative administration in repeated cycles, has also been proposed to reduce the recurrence of benign epithelial tumors, particularly inverted papillomas [4,8].

## 9. Emerging Developments

Advances in surgical techniques, molecular diagnostics, and targeted therapies may influence future management of LDS tumors.

An endoscopic approach alone for full resection has been reported for early-stage tumors [5], and navigation assistance may allow en bloc resection of the lacrimal sac and duct through a transnasal approach [5,6]. However, to ensure adequate oncologic margins, the use of external or combined approaches may be preferred for most malignancies [29]. Novel techniques, such as a “prelacrimal recess” approach (Figure 5E), might provide improved access to the lacrimal sac and maxillary sinus whilst preserving anatomical structures, such as the inferior turbinate [7].

Molecular profiling has identified possible targets for treatment of some LDS tumors. Human Epidermal Growth Factor Receptor 2 (HER2) amplification might support the future use of HER2-directed therapies such as trastuzumab and/or tyrosine kinase inhibitors [30]. Advances in melanoma biology have similarly identified actionable biomarkers, including NRAS and BRAF mutations and PD-L1 expression, informing the use of targeted therapies, including BRAF/MEK inhibition and immune checkpoint inhibitors [31,32].

Human papillomaviruses (HPVs) are implicated in some lacrimal sac carcinomas, particularly non-keratinizing, transitional-type, and papillary squamous cell carcinomas—with high-risk HPV (notably HPV-16) detected in up to 80% of cases [24]. HPV-positive SCCs exhibit molecular features similar to other HPV-driven head and neck cancers, including p16 overexpression, wild-type TP53, and frequent PI3K/AKT pathway alterations [10,31,33,34,35]. Both low-risk (HPV-6, HPV-11) and high-risk HPV strains are often identified in lacrimal sac papillomas, particularly the endophytic subtype [14]. HPV-positive tumors show increased sensitivity to chemoradiotherapy, and such neoadjuvant therapy may facilitate lesser surgery in patients with extensive disease, who otherwise would have undergone major craniocervical resection, with or without maxillectomy [33,34]. Identifying molecular genomic profiles in HPV-positive LDS tumors may help identify future targeted therapies [35].

While proton beam therapy is established for orbital and LDS tumors, carbon ion radiotherapy (CIRT) may be considered for aggressive or radioresistant malignancies. Its higher linear energy transfer induces more complex and less repairable DNA damage, which may enhance treatment efficacy in resistant tumors, such as adenoid cystic carcinoma [36].

## 10. Prognosis

Although benign, inverted papillomas carry a risk for malignant transformation and should therefore be monitored long-term for recurrence. The reported 5-year recurrence rate is 10–40%, with minimal difference between endoscopic or external techniques, and the average time to recurrence is 4.5 years (ranging from 6 months to 10 years) [8,37,38]. The rate of malignant transformation is 10–15%, with the timeframe being variable and unpredictable. Follow-up imaging is recommended for a minimum of 5–7 years, or longer in higher-risk patients [37,38].

Recurrence rates for epithelial malignancies range from 11% to 66%, with a 5-year survival rate ranging from 61% to 88% [2,10,29,39]. Prognosis is influenced primarily by tumor stage and extent of disease. Metastatic spread has been reported in up to 35% of patients, while higher T-stage and positive lymph node status are associated with poorer survival [10]. Although histological subtype has not been validated as an independent prognostic factor, non-keratinizing squamous cell carcinoma and lymphoepithelial carcinoma appear to have higher metastatic rates [10]. Globe preservation is achievable in approximately 70–100% of patients, with most retaining functional vision, while orbital exenteration appears to offer no clear survival advantage despite its substantial morbidity [40,41]. Recurrence rates for lacrimal sac melanomas range between 17% and 27%, with the rate of distant metastasis being around 40% [11,42,43]. The overall 5-year survival rate for melanoma is about 20% [11,41,42].

## 11. Conclusions

Tumors of the lacrimal drainage system are rare and often mimic benign lacrimal disease, contributing to delayed diagnosis and complex management. Early recognition, appropriate imaging, and timely biopsy are essential for optimal care. Complete surgical excision remains the mainstay of treatment, while advances in endoscopic surgery, radiotherapy, immunotherapy, and molecular profiling are expanding opportunities for globe preservation and individualized treatment. Multidisciplinary management and long-term surveillance remain critical to improving oncologic and functional outcomes.

## Figures and Tables

**Table 1:** Histopathological classification for tumors involving the lacrimal drainage system.

Tumor Subgroup	Benign	Malignant
Epithelial	Papilloma Oncocytoma Adenoma Cylindroma	Squamous cell carcinoma (SCC) Non-keratinizing SCC * Mucoepidermoid carcinoma Adenocarcinoma Adenoid cystic carcinoma Oncocytic adenocarcinoma Lymphoepithelial carcinoma Primary small cell carcinoma Sebaceous carcinoma Merkel cell carcinoma
Mesenchymal	Solitary fibrous tumor Haemangioma Fibroma Lipoma Angiofibroma Leiomyoma Juvenile xanthogranuloma Osteoma	Kaposi sarcoma Rhabdomyosarcoma
Lymphoproliferative	Reactive lymphoid hyperplasia	Diffuse large B-cell lymphoma Extranodal marginal zone B-cell lymphoma Unclassified B-cell lymphomas Small cell lymphoma NK/T cell lymphoma Myeloid sarcoma
Melanocytic	Benign naevus Benign melanosis	Malignant melanoma
Neural	Neurofibroma Neurilemmoma	

* Non-keratinizing SCC includes transitional cells and poorly differentiated and undifferentiated carcinomas.

**Table 2:** Non-neoplastic differential diagnoses of lacrimal drainage system tumors (LDS).

Category	Differential Diagnoses
Inflammatory/Infectious	Chronic dacryocystitis, inflammatory granulation tissue, dacryocystocele/mucocele, actinomycosis, tuberculosis, fungal dacryocystitis, fungal granuloma
Granulomatous/Autoimmune	Sarcoidosis, granulomatosis with polyangiitis, IgG4-related disease, xanthogranuloma
Idiopathic Inflammatory	Non-specific orbital inflammatory disease (sclerosing and non-sclerosing variants) involving LDS
Cystic/Developmental	Epidermoid cyst, dermoid cyst, lacrimal sac diverticulum, encephalocele
Vascular/Other	Capillary hemangioma, cavernous venous malformation, amyloidosis

## Data Availability

No new data were created or analyzed in this study.
